# A label-free and portable graphene FET aptasensor for children blood lead detection

**DOI:** 10.1038/srep21711

**Published:** 2016-02-24

**Authors:** Chenyu Wang, Xinyi Cui, Ying Li, Hongbo Li, Lei Huang, Jun Bi, Jun Luo, Lena Q. Ma, Wei Zhou, Yi Cao, Baigeng Wang, Feng Miao

**Affiliations:** 1 National Laboratory of Solid State Microstructures, School of Physics, Collaborative Innovation Center of Advanced Microstructures, Nanjing University, Nanjing 210093, China; 2 State Key Laboratory of Pollution Control and Resource Reuse, School of the Environment, Nanjing University, Nanjing 210046, China; 3 Jiangsu Engineering Technology Research Centre of Environmental Cleaning Materials, Jiangsu Key Laboratory of Atmospheric Environment Monitoring and Pollution Control, Jiangsu Joint Laboratory of Atmospheric Pollution Control, Collaborative Innovation Center of Atmospheric Environment and Equipment Technology, School of Environmental Science and Engineering, Nanjing University of Information Science & Technology, 219 Ningliu Road, Nanjing, Jiangsu 210044, China

## Abstract

Lead is a cumulative toxicant, which can induce severe health issues, especially in children’s case due to their immature nervous system. While realizing large-scale monitoring of children blood lead remains challenging by utilizing traditional methods, it is highly desirable to search for alternative techniques or novel sensing materials. Here we report a label-free and portable aptasensor based on graphene field effect transistor (FET) for effective children blood lead detection. With standard solutions of different Pb^2+^ concentrations, we obtained a dose-response curve and a detection limitation below 37.5 ng/L, which is three orders lower than the safe blood lead level (100 μg/L). The devices also showed excellent selectivity over other metal cations such as, Na^+^, K^+^, Mg^2+^, and Ca^2+^, suggesting the capability of working in a complex sample matrix. We further successfully demonstrated the detection of Pb^2+^ ions in real blood samples from children by using our aptasensors, and explored their potential applications for quantification. Our results underscore such graphene FET aptasensors for future applications on fast detection of heavy metal ions for health monitoring and disease diagnostics.

Lead (Pb) is a widely distributed contaminant due to industrial and mining activities, coal combustion, and use of leaded paints/gasoline^1^,^2^,^3^. Mounting evidence showed that Pb can do harm to numerous human body systems, such as the neurological, haematological, gastrointestinal, cardiovascular and renal systems^4^. For children, Pb is more dangerous due to their immature nervous system. Deleterious effects on children’s brain functions, including low intelligence and behavioral development problems, have been diagnosed when blood lead level (BLL) is above 100 μg/L^5^. When BLL is below 75 μg/L, the reduction of children’s intelligence quotient (IQ) was reported to be associated with as well^5^, indicating that there is no safe level for children blood Pb. Thus, while numbers of legislative efforts have been undertaken to reduce the Pb exposure, it is of key importance to develop effective detection techniques for large-scale monitoring of children blood Pb.

Some basic criteria to realize large-scale monitoring of blood Pb include good selectivity, high sensitivity, superior portability, fast response and low cost. However, due to the complicated matrix of blood, it is highly challenging to develop a detection technique, which combines all these required features. For example, some widely-used instruments in laboratories such as atomic absorption spectrometry (AAS) and inductively coupled plasma mass spectrometry (ICP-MS) have shown good selectivity and high sensitivity, but they are quite expensive and cumbersome^6^,^7^. The electrochemical approaches^7^,^8^,^9^,^10^,^11^,^12^,^13^,^14^. such as anodic stripping voltammetry (ASV), are simple and economical, but need improvement on both sensitivity and selectivity. The application of other approaches, fluorescent and colorimetric methods for instance, are limited by slow response time^15^,^16^,^17^,^18^,^19^,^20^,^21^,^22^. Alternative techniques or novel sensing materials are highly demanded to meet all the aforementioned criteria and achieve on-site, sensitive and fast detection of children blood lead.

Graphene, the world’s thinnest conductive and elastic material, emerges as an ideal candidate. As a prototype two-dimensional (2D) material, graphene is easy to be functionalized and highly sensitive to environment, making it suitable to detect various objects, such as proteins, DNA, gas atoms and heavy metal ions^19^,^23^,^24^,^25^,^26^,^27^,^28^,^29^. For example, graphene liquid-gate transistor-type aptamer sensor has shown great potential and attracted much attention in the aspect of biosensor. Furthermore, with recent tremendous progresses on economical wafer-scale growth, graphene has been widely recognized as a top candidate for ultra-scaled portable and flexible electronics.

In this work, we report a label-free and portable aptasensor based on graphene FET for effective children blood Pb detection. With Tris-HCl solutions of different Pb^2+^concentrations, we obtained a detection limitation below 37.5 ng/L. Excellent selectivity to was also demonstrated over other metal cations Na^+^, K^+^, Mg^2+^, and Ca^2+^, suggesting the devices’ capability of working in a complex sample matrix. Furthermore, we successfully demonstrated the detection of Pb^2+^ ions in real blood samples from children by using our aptasensors, and explored their potential applications for quantification.

## Results and Discussions

**Figure 1:**
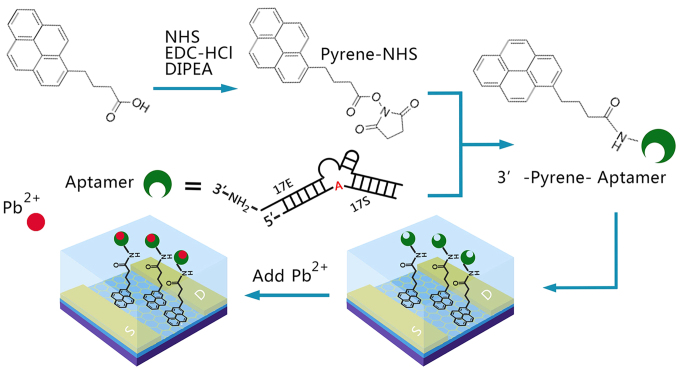
Illustrative scheme of the preparation of graphene field effect transistor (FET). Details are in the Supplementary Information .

Figure 1 shows the process to prepare the graphene FET aptasensor. Many aptamers have been selected for Pb^2+^ detection, including G-quadruplex^15^,^17^,^18^, Thrombin binding aptamer (TBA)^20^,^30^, and 8–17 DNAzyme^19^,^21^,^29^. We chose 8–17 DNAzyme as the Pb sensing aptamer because of its high Pb^2+^ binding affinity and selectivity. The original 8–17 DNAzyme comprises of an enzyme strand (17E), which cleaves the RNA base on the substrate (17S) strand at the cleavage site upon binding to Pb^2+^. Typically, 8–17 DNAzyme based Pb^2+^ sensors relied on the release of cleaved 17S strand for signaling^19^,^21^,^29^. In our devices, we observed that Pb^2+^ binding alone (by replacing the cleavable site rebonucleotide “A” to uncleavable deoxyribonucleotide “A” of the 8–17 DNAzyme) could give rise to significant signal changes with no need for the dissociation of the cleaved 17S strand. This could potentially improve the response speed of the 8–17 DNAzyme based Pb^2+^ sensors^31^. Therefore, we modified the RNA base, adenine, in 17S to a DNA base, making the substrate uncleavable yet retaining the Pb^2+^ binding capability of the DNAzyme. To ensure strong and specific binding of the aptamers to the graphene surface, we introduced a pyrene group to the 5′-end of 17E. This allows the aptamers to be anchored to the graphene surface of the devices through π-π stacking interactions. Moreover, this could successfully avoid the non-specific adsorption and denaturation of 8–17 DNAzyme on the graphene surface, which was reported to be a problem for many graphene based sensors^29^. The detailed procedures for the synthesis of the modified 8–17 DNAzyme can be found in the Supplementary Information. To functionalize the devices, we directly added the modified 8–17 DNAzyme aptamer to the graphene surface of the device, which was pre-wetted by Tris-HCl buffer (0.5 M, pH = 7.2). Then, the aptamer solutions were kept on the graphene surface for ~10 min to ensure the complete adsorption. The unbound aptamer was then removed by rinsing the surface with Tris-HCl buffer for 3 times. After being functionalized with aptamers, the graphene devices were ready for Pb^2+^ sensing. Such approaches have also been demonstrated to be valid to detect other heavy metal ions, such as Hg^2+ ^ 23.

**Figure 2:**
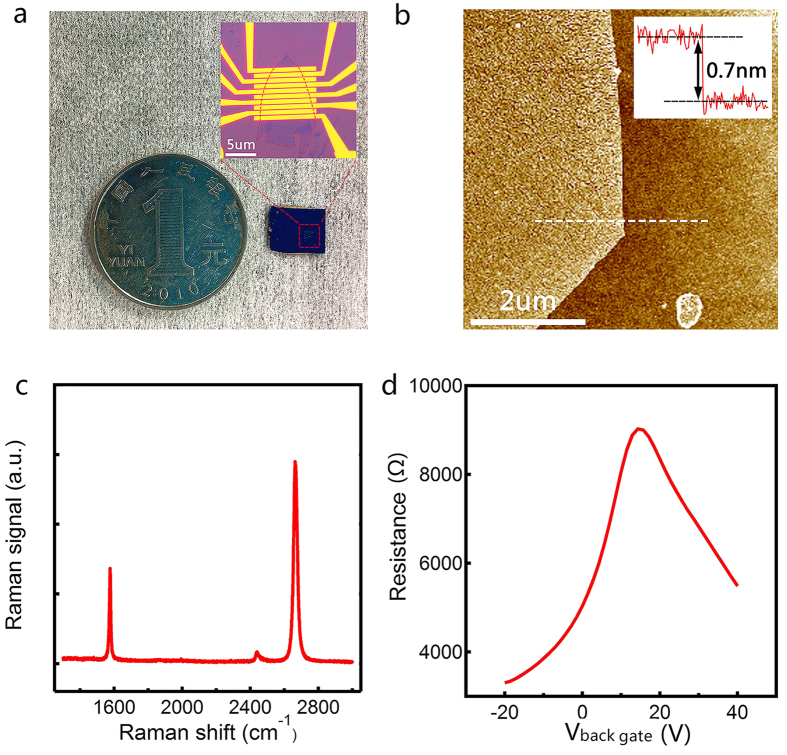
(**a**) The optical microscopy pictures of a graphene device on Si wafer and a coin for comparison; (**b**) The AFM image and thickness of a monolayer graphene flake (~0.7 nm); (**c**) The Raman spectrum of a monolayer graphene device. (**d**) Resistance-back gate voltage curve of a typical graphene FET device.

The optical microscopic image of a typical graphene device is shown in Fig. 2a. The typical size of the devices is in micron scale, with the potential of even scaling down, suggesting good portability which is highly important in large-scale on-site BLL measurements. The mechanically-exfoliated graphene flakes on standard Si wafers (covered by 300 nm-thick SiO_2_) were chosen for our studies due to higher quality, while CVD (Chemical Vapor Deposition) grown graphene is believed to behave similarly. Shadow mask alignment or electron beam lithography followed by electron beam evaporation was applied to pattern the electrodes, which consist of 5 nm Ti and 50 nm Au, covered by an extra layer of 15 nm SiO_2_ to protect electrodes from electrochemical reactions (details can be found in Materials and Methods). The atomic force microscopy (AFM) image of a single layer graphene flake is presented in Fig. 2b, where the thickness of the flake was determined to be about 0.7 nm. Raman spectroscopy was also used to confirm the layer number of graphene flakes. In the Raman spectrum shown in Fig. 2c, the G-peak (~1600 ) and 2D-peak (~2700 ) are clearly resolved with strong intensity and sharp shape of 2D peak, indicating that the graphene flake is monolayer. To avoid possible surface contaminations, all devices were thermal-annealed (Ar 1.5 ccm and H_2_ 250 sccm, 300K) before electrical measurements were performed.

Figure 2d presents the back gate (V_back gate_)-dependent resistance measurement results of a typical graphene FET device, showing the ambipolar field-effect characteristics. Both the carrier type and density of graphene can be modulated by V_back gate_. In Fig. 2d, the maximum resistance (or the minimal conductance) point corresponds to the charge neutrality point V_cnp_ (or the Dirac point in the case of monolayer graphene), indicating how the graphene flake is intrinsically doped, i.e. p/n doped when V_cnp_ is positive/negative or non-doped when V_cnp_ is equal to zero. For this particular device, V_cnp_ is around 18V, suggesting it is intrinsically p-doped. The feature of being sensitive to surrounding environment renders graphene an ideal sensing material for detecting charged objects. With the presence of certain charged environment, the charge doping level (or Fermi level) of graphene will be changed, resulting in a shift of V_cnp_, or the resistance-V_back gate_ curve.

**Figure 3:**
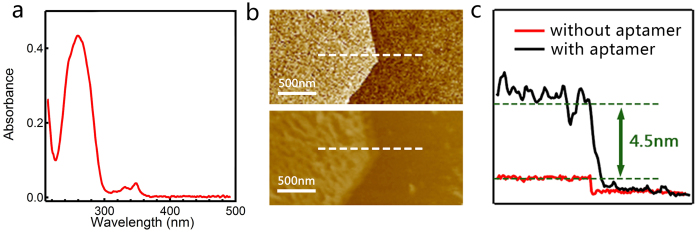
(**a**) The fluorescence absorbance of 8–17 DNAzyme and pyrene combination; (**b**) The AFM microphotographs of graphene thickness before and after functionalization. The top is before functionalization and the bottom is after functionalization. (**c**) The change of thickness before and after functionalization.

We first confirmed the successful linking of pyrene to the 5′ of 17E, as supported by the UV-Vis spectrum shown in Fig. 3a. The signals in the range of 300 nm to 400 nm correspond to the adsorption of pyrene and the signals around 280 nm correspond to the adsorption from nucleotides. Based on their extinction coefficients, the conjugation efficiency is ~100%. In order to confirm the aptamers were successfully attached to the graphene surfaces, we compared the height of the graphene layer before and after the functionalization. As shown in the AFM images in Fig. 3b,c, the height of the graphene layer increased by ~4.5 nm upon the functionalization with the aptamers, which is consistent with the size of 8–17 DNAzyme. Because the structure of the aptamers is flexible, the height of the functionalized surface showed larger fluctuations than that of the pristine graphene flake. Taken together, these characteristics indicated the successful realization of the device design shown in Fig. 1.

**Figure 4:**
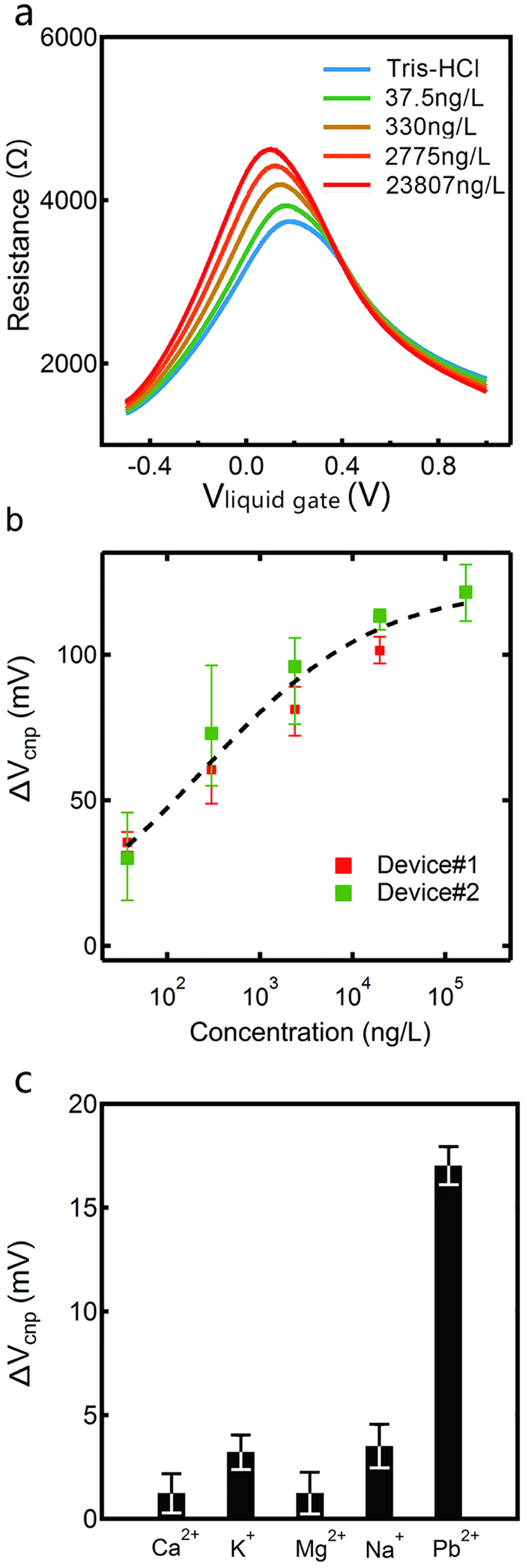
(**a**) The resistance-V_liquid gate_ curves of aptasensor in tris-HCl solution with five different Pb^2+^ concentrations (0–23807 ng/L); (**b**) The measured ∆V_cnp_ versus concentration (in logarithmic scale). The black dotted line corresponds to the fitting results by using a lognormal function (y=y0+Aexp{−[ln(x/x0)⁄width]_2_}, with y0 = −130.47, A = 103.62, x0 = 5.84, width = 6.31); (**c**) The selectivity of the aptasensor in solutions of Na^+^, K^+^, Mg^2+^, and Ca^2+^, and at a concentration of 0.1 M/L while Pb^2+^ at a concentration of 0.5 nM/L.

To examine the performance of our graphene aptasensors for detection, we used 10 mg/L solution diluted with Tris-HCl to obtain standard solutions with different concentrations. The devices were immersed in the 0.5 M/L Tris-HCl solution during test to simulate human body fluid environment and build the ion liquid gate type of FETs (see Materials and Methods for details). After adding standard solutions with different concentrations, we measured the resistance-V_liquid gate_ curves within 2 minutes, which minimized the effect of solution evaporation. The pH of standard solutions was kept at neutral to avoid false response. At least two replicates were conducted for each concentration and the relative standard deviations between replicates were 2.4–15.7%, indicating good reproducibility of our measurement. The test results of standard solutions with four concentrations (i.e., 37.5 ng/L, 330 ng/L, 2775 ng/L, 23807 ng/L) are shown in Fig. 4a. Before adding solutions, the V_cnp_ was around 0.2 V, indicating the p-doping nature of the device in Tris-HCl environment. After adding standard solution of 37.5 ng/L, we clearly observed left shifts of the curve, i.e. smaller V_cnp_. For higher concentrations, the resistance-V_liquid gate_ shifted further left and V_cnp_ became smaller.

Such results indicate that our graphene aptasensors are effective for detecting at different concentrations. The detection mechanism can be explained by a simple picture that, in solutions cause electron doping of the graphene (which is intrinsically p-doped) by approaching its surface, which leads to a left shift of the transfer curves or a smaller V_cnp_. For solutions with higher concentration, larger doping of electrons induces larger shift of V_cnp_, which can be defined by ∆V_cnp_^26^,^28^,^29^ and potentially used as an effective parameter to characterize ion concentrations. We tested multiple devices with similar response observed. In Fig. 4b, we plotted the measured ∆V_cnp_ versus concentration (in logarithmic scale) of the tested standard solutions from two representative devices (with additional dataset plotted in Supplementary Information Fig. S2). A lognormal fitting was performed to obtain a calibration curve for quantification. Based on our measurement, the lowest concentration our graphene aptasensor detected was 37.5 ng/L, which was approximately one thousandth of the safety line (100 ug/L) for blood Pb. This demonstrated that our aptasensor can work well for samples with much lower than safe level.

To study the selectivity of the aptasensor toward Pb^2+^, we measured the response of other commonly found ions in blood, including Na^+^, K^+^, Mg^2+^ and Ca^2+^ at a concentration of 0.1 M/L. As shown in Fig. 4c, when solutions containing these metal ions were added to the same device, the observed ∆V_cnp_ was much smaller than that from Pb^2+^ at a significantly lower concentration (0.5 nM/L). Therefore, our device can be used to measure real blood samples with no interference from other metal cations in blood. Such a great selectivity can be attributed to the preferential binding of 8–17 DNAzyme to Pb^2+^ over other metal ions. These results also suggested that functionalization of 8–17 DNAzyme to the graphene surface did not affect its Pb^2+^ binding affinity and selectivity.

**Figure 5:**
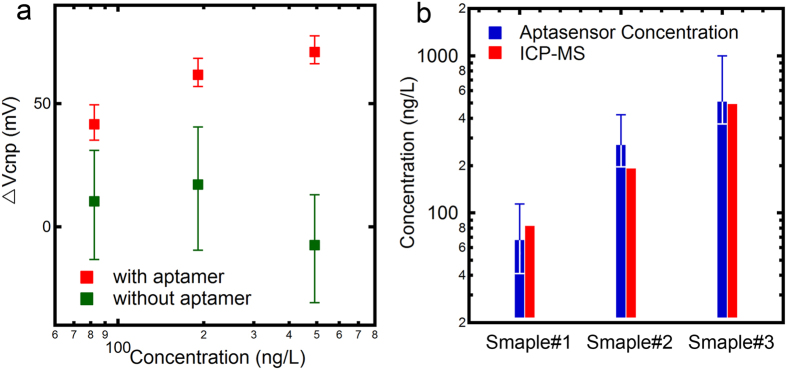
(**a**) The detection of Pb^2+^ ions in three real blood samples with different concentrations and the comparison of control experiments on another non-functionalized device; (**b**) The comparison between aptasensor measured concentration and ICP-MS measured concentration.

To the best of our knowledge, although a few graphene FET aptasensor has been reported, none of them was used for the measurement of Pb level in real blood sample. To address this challenging issue, we tested the feasibility of our device to measure BLL in real children blood samples. Three children blood samples were collected from town of Zhuhang in Nantong (Jiangsu Province, China), which is an industrial town with intensive wire rope production. Emission of Pb-containing smoke and dust during the wire rope manufacturing process result in elevated levels of Pb in various environmental matrix of this area. Several children living in a nearby residential district were diagnosed with hyperactivity and emotional problems owing to their elevated BLL higher than 100 μg/L^32^. The concentrations measured by ICP-MS were 82.4, 191.1, and 491.6 ng/L for sample 1, 2 and 3, respectively. The real samples were pretreated before tested by our device (see Materials and Methods for details). Briefly, the blood samples were digested with nitric acid to dissolve cells and proteins, and the solution was neutralized by NaOH (1M/L). The three samples were added and tested on one same device. Figure 5a shows that ∆V_cnp_ increased with Pb^2+^ concentration. In order to rule out possible background disturbance in real samples, a parallel test was performed with another non-aptamer device, with results shown in Fig. 5a as well. There was no significant change while concentration increased, indicating that in real sample can’t lead to a shift of V_cnp_ on the bare graphene.

We further explored the potential application of our aptasensor devices on Pb quantification in real blood samples. The results from the standard solutions (as shown in Fig. 4b) were used as the calibration curve, and the concentration can be derived according to the measured signals of ∆V_cnp_. We defined these concentrations as aptasensor concentrations, which were plotted together with ICP-MS measured concentrations for comparison in Fig. 5b. Good agreement was obtained, indicating that, with further improvement on material quality and device uniformity in future, our aptasensor devices could offer an alternative approach to measuring BLL in real blood samples in a very convenient and accurate way.

## Conclusions

In summary, we demonstrated a label-free and portable aptasensor based on graphene FET with the capability of working in the real blood sample. The aptasensor consisted of graphene with the surface functionalized by 8–17 DNAzyme, yielding excellent selectivity to Pb^2+^. In standard solutions with different Pb^2+^ concentrations, we measured a detection limitation below 37.5 ng/L, which is much lower than the safety line regulated for children blood lead. We further successfully demonstrated the detection of Pb^2+^ ions in real blood samples from children, and explored their potential applications on determining the concentrations. Our results suggest that such graphene FET aptasensors could be widely used for fast detection of heavy metal ions in human health monitoring and disease diagnostics.

## Methods

### Materials and Devices

The kish graphite for mechanical exfoliation was purchased from Covalent Material Co. (Tokyo, Japan). 17E and 17S was separately synthesized by Genscript Biotechnology Co. (Nanjing, China) and dissolved in deionized water (4 μM/L). Our real samples of children blood were collected from Zhuhang, Nantong, Jiangsu Province, China. The collection of blood samples and the related experiments performed in this study were approved by the center for disease control and prevention of Changshu, China, and the methods were carried out in accordance with the guidelines established by the center. The informed consent was obtained from parents of these children. A conventional electron-beam lithography process followed by standard electron-beam evaporation of metal electrodes (typically 5 nm Ti/40 nm Au) was used to fabricate graphene FET devices.

### Functionalization of Graphene

17E and 17S were synthesized by Genscript Biotechnology Co. (Nanjing, China). The functionalization consists of two steps: firstly, we dropped the 17E solution (4 μM/L) on the devices, and waited for 30 minutes to ensure good attachment; Secondly, we dropped the 17S to form 8–17 DNAzyme on the surface of graphene. The devices were washed with deionized water to remove the aptamer residue after each step.

### Pretreatment of Real Samples and Measurement with ICP-MS

The children blood was digested according to U.S. EPA Method 3050B through a Hot Block digestion system (Environmental Express, Mt. Pleasant, SC). Briefly, 0.5 mL of blood was initially digested with 10 mL of concentrated HNO_3_ in a graphite digestion system at 105 °C for 5 h, and followed by the addition of 1 mL of 30% H_2_O_2_. The digestion was continued to reach near dryness, which was diluted to 10 mL with Milli-Q water. Lead concentration in the digestion solution was measured using ICP-MS. We measured it with ICP-MS (NexION300X, PerkinElmer). During ICP-MS analysis, indium isotope (^114^In) was used as internal standard to test the instrument performance. Before tested with our biosensor, the digested samples were neutralized by NaOH (1M/L).

### Electrical Measurement

NI USB-6251 (National Instruments. Austin, USA) was used as a voltage source and DL 1211 (DL instruments. New York, USA) was used as a current amplifier. The doped silicon layer underneath the SiO_2_ layer of the wafers was used as the back gate. For the ion liquid gate type of FET devices, a gold wire was inserted into the solution as the liquid gate electrode. We applied a constant bias = 0.1 V while sweeping the back/liquid gate voltage forward at a speed of 0.01 V/s. There was a one-minute internal between each gate voltage sweep to ensure a stable state. For measurements of real samples, an initial signal offset was adjusted by adding a highly-diluted pretreated sample with concentration (~5 ng/L). The error bars arose from the upper and lower limit of multi-measurements operated in a certain concentration.

## Additional Information

**How to cite this article**: Wang, C. *et al.* A label-free and portable graphene FET aptasensor for children blood lead detection. *Sci. Rep.* **6**, 21711; doi: 10.1038/srep21711 (2016).

## Supplementary Material

Supplementary Information

## References

[b1] Lei M., Tie B., Williams P. N., Zheng Y. & Huang Y. Arsenic, Cadmium, and Lead Pollution and Uptake by Rice (Oryza Sativa L.) Grown in Greenhouse. J Soil Sediment. 11, 115–123 (2011).

[b2] Hassan W. & David J. Effect of Lead Pollution On Soil Microbiological Index Under Spinach (Spinacia Oleracea L.) Cultivation. J Soil Sediment. 14, 44–59 (2014).

[b3] Rodríguez Martín J. A. *et al.* Effect of Mine Tailing On the Spatial Variability of Soil Nematodes From Lead Pollution in La Union (Spain). Sci Total Environ. 473–474, 518–529 (2014). 10.1016/j.scitotenv.2013.12.075 24394364

[b4] World Health Organization. *Exposure to* Lead: A *Major Public Health Concern* (2010). Available at: http://www.who.int/ipcs/features/lead.pdf. (Accessed: 17th January 2016).

[b5] P L. B., R H. & J K. Low-Level Environmental Lead Exposure and Children’s Intellectual Function: An International Pooled Analysis. Environ Health Persp. 113(**7**), 894–899 (2005). 10.1289/ehp.7688 PMC1257652 16002379

[b6] World Health Organization. *Brief Guide to Analytical Methods for Measuring Lead in Blood* (2011). Available at: http://apps.who.int/iris/bitstream/10665/77912/1/9789241502139_eng.pdf. (Accessed: 17th January 2016).

[b7] Deibler K. & Basu P. Continuing Issues with Lead: Recent Advances in Detection. Eur J Inorg Chem. 2013, 1086–1096 (2013). 25089117 10.1002/ejic.201200997 PMC4116340

[b8] Arduini F., Calvo J. Q., Palleschi G., Moscone D. & Amine A. Bismuth-Modified Electrodes for Lead Detection. Trac-Trend Anal Chem 29, 1295–1304 (2010).

[b9] Laschi S., Palchetti I. & Mascini M. Gold-Based Screen-Printed Sensor for Detection of Trace Lead. Sensors and Actuators B: Chemical 114, 460–465 (2006).

[b10] Kruusma J., Banks C. & Compton R. Mercury-Free Sono-Electroanalytical Detection of Lead in Human Blood by Use of Bismuth-Film-Modified Boron-Doped Diamond Electrodes. Anal Bioanal Chem. 379, (2004). 10.1007/s00216-004-2639-5 15185062

[b11] Yantasee W., Lin Y., Zemanian T. S. & Fryxell G. E. Voltammetric Detection of Lead(ii) and Mercury(ii) Using a Carbon Paste Electrode Modified with Thiol Self-Assembled Monolayer On Mesoporous Silica (Samms). The Analyst. 128, 467–472 (2003). 12790199 10.1039/b300467h

[b12] Dragoe D. *et al.* Detection of Trace Levels of Pb^2+^ in Tap Water at Boron-Doped Diamond Electrodes with Anodic Stripping Voltammetry. Electrochim Acta. 51, 2437–2441 (2006).

[b13] Wei Y. *et al.* Sno_2_ Reduced Graphene Oxide Nanocomposite for the Simultaneous Electrochemical Detection of Cadmium(Ii), Lead(Ii), Copper(Ii), and Mercury(Ii): An Interesting Favorable Mutual Interference. J Phys Chem C. 116, 1034–1041 (2012).

[b14] Xiao Y., Rowe A. A. & Plaxco K. W. Electrochemical Detection of Parts-Per-Billion Lead Via an Electrode-Bound Dnazyme Assembly. J Am Chem Soc. 129, 262–263 (2007). 17212391 10.1021/ja067278x

[b15] Li T., Wang E. & Dong S. Lead(Ii)-Induced Allosteric G-Quadruplex Dnazyme as a Colorimetric and Chemiluminescence Sensor for Highly Sensitive and Selective Pb^2+^ Detection. Anal Chem. 82, 1515–1520 (2010). 20095579 10.1021/ac902638v

[b16] Fu X. *et al.* “Turn-On” Fluorescence Detection of Lead Ions Based On Accelerated Leaching of Gold Nanoparticles On the Surface of Graphene. Acs Appl Mater Inter. 4, 1080–1086 (2012). 10.1021/am201711j 22264012

[b17] Li M., Zhou X., Guo S. & Wu N. Detection of Lead (Ii) with a “Turn-On” Fluorescent Biosensor Based On Energy Transfer From Cdse/Zns Quantum Dots to Graphene Oxide. Biosens Bioelectron. 43, 69–74 (2013). 23277342 10.1016/j.bios.2012.11.039

[b18] Li T., Dong S. & Wang E. A Lead(Ii)-Driven Dna Molecular Device for Turn-On Fluorescence Detection of Lead(Ii) Ion with High Selectivity and Sensitivity. J Am Chem Soc. 132, 13156–13157 (2010). 20822179 10.1021/ja105849m

[b19] Wen Y. *et al.* Metal Ion-Modulated Graphene-Dnazyme Interactions: Design of a Nanoprobe for Fluorescent Detection of Lead (ii) Ions with High Sensitivity, Selectivity and Tunable Dynamic Range. Chem Commun. 47, 6278 (2011). 10.1039/c1cc11486g 21503363

[b20] Liu C., Huang C. & Chang H. Highly Selective Dna-Based Sensor for Lead(Ii) and Mercury(Ii) Ions. Anal Chem. 81, 2383–2387 (2009). 19219985 10.1021/ac8022185

[b21] Wang Z., Lee J. H. & Lu Y. Label-Free Colorimetric Detection of Lead Ions with a Nanomolar Detection Limit and Tunable Dynamic Range by Using Gold Nanoparticles and Dnazyme. Adv Mater. 20, 3263–3267 (2008).

[b22] Zhao X. *et al.* Graphene–Dnazyme Based Biosensor for Amplified Fluorescence “Turn-On” Detection of Pb^2+^ with a High Selectivity. Anal Chem. 83, 5062–5066 (2011). 21639104 10.1021/ac200843x

[b23] An J. H., Park S. J., Kwon O. S., Bae J. & Jang J. High-Performance Flexible Graphene Aptasensor for Mercury Detection in Mussels. Acs Nano. 7, 10563–10571 (2013). 24279823 10.1021/nn402702w

[b24] Ohno Y., Maehashi K. & Matsumoto K. Label-Free Biosensors Based On Aptamer-Modified Graphene Field-Effect Transistors. J Am Chem Soc. 132, 18012–18013 (2010). 21128665 10.1021/ja108127r

[b25] Joshi R. K. *et al.* Precise and Ultrafast Molecular Sieving through Graphene Oxide Membranes. Science. 343, 752–754 (2014). 24531966 10.1126/science.1245711

[b26] Kim D. *et al.* Reduced Graphene Oxide Field-Effect Transistor for Label-Free Femtomolar Protein Detection. Biosens Bioelectron. 41, 621–626 (2013). 23107386 10.1016/j.bios.2012.09.040

[b27] Park S. J. *et al.* Ultrasensitive Flexible Graphene Based Field-Effect Transistor (Fet)-Type Bioelectronic Nose. Nano Lett. 12, 5082–5090 (2012). 22962838 10.1021/nl301714x

[b28] Cai B. *et al.* Ultrasensitive Label-Free Detection of Pna–Dna Hybridization by Reduced Graphene Oxide Field-Effect Transistor Biosensor. Acs Nano. 8, 2632–2638 (2014). 24528470 10.1021/nn4063424

[b29] Wen Y. *et al.* The Electrical Detection of Lead Ions Using Gold-Nanoparticle- And Dnazyme-Functionalized Graphene Device. Adv Healthc Mater. 2, 271–274 (2013). 23184414 10.1002/adhm.201200220

[b30] Xu K. *et al.* Graphene- And Aptamer-Based Electrochemical Biosensor. Nanotechnology (2014). 10.1088/0957-4484/25/20/205501 24785149

[b31] Kim H., Rasnik I., Liu J., Ha T. & Lu Y. Dissecting Metal Ion–Dependent Folding and Catalysis of a Single Dnazyme. Nat Chem Biol. 3, 763–768 (2007). 17965708 10.1038/nchembio.2007.45 PMC4376948

[b32] Li H., Chen K., Juhasz A. L., Huang L. & Ma L. Q. Childhood Lead Exposure in an Industrial Town in China: Coupling Stable Isotope Ratios with Bioaccessible Lead. Environ Sci Technol. 49, 5080–5087 (2015). 25803404 10.1021/es5060622

