# Supplementary material for: A label-free and portable graphene FET aptasensor for children blood lead detection
Source: Sci Rep. 2016 Feb 24;6:21711. doi: 10.1038/srep21711 (PMC4764843; doi:10.1038/srep21711)
Supplement: Supplementary Information [file srep21711-s1.pdf]

Supplementary Information for

A label-free and portable graphene FET aptasensor for childhood blood lead detection

Chenyu Wang[1], *Xinyi Cui**[2], *Ying Li**[3], *Hongbo Li*[2], *Lei Huang*[2], *Jun Bi*[2], *Jun Luo*[2], *Lena Q. Ma*[2], *Wei Zhou*[1], *Yi Cao*[1], *Baigeng Wang*[1], *Feng Miao**[1]

Preparation of pyrene-derivatized aptamer 17E

Pyrenebutyric acid (120 mg, 0.416 mmol), *N*-hydroxyl succimide (53 mg, 0.458 mmol) and triethylamine (150 μL, 1.05 mmol) were dissolved in dichloromethane (20 mL). The resulted solution was added with 1-ethyl-3-(3-dimethylaminopropyl)carbodiimide hydrochloride (103 mg, 0.540 mmol). The reaction was stirred at room temperature for 24 hr and then concentrated *in vacuo*. The residue was subject to silica gel flash chromatography to give pale red solid as the desired product of pyrene-NHS (78 mg, Yield: 48.6 %). ^{1}H NMR (500 MHz, $CDCl_3$ with TMS as the internal standard r.t.): *δ(ppm)* 2.34 (2 H, *quintet*, J = 7.43 Hz), 2.77 (2 H, *t*, J = 7.10 Hz), 2.88 (4 H, *s*), 3.51 (2 H, *t*, J = 7.72 Hz), 7.91 (1 H, *d*, J = 7.76 Hz), 8.03 (3 H, *m*), 8.18 (4 H, *m*), 8.32 (1 H, *d*, J = 9.22 Hz).

3'-Amino derivatized aptamer 17E (sequence: 5'-GACATCTCTTCTCCGAGCCGGTCGAAA TAGTGAGTTTTTT-3', 16.8 nmol) was first dissolved in autoclaved ddH_2O (300 μL) and 8% cetyl trimethylammonium bromide (CTAB, 5 μL) was added to the solution to precipitate the oligonucleotide.

The mixture was subject to centrifuge at 13k rpm for 10 min. Another 3 μL of the CTAB solution was added to further precipitate the oligonucleotide (generally the solution did not turn cloudy this time). The mixture was again centrifuged at 13k rpm for 10 min. The supernatant was removed, and the pellet was washed with autoclaved ddH_2O (1 mL) three times, dissolved in anhydrous ethanol (600 μL) and dried in a Speed Vac for 1.5 hr. Then the oligonucleotide pellet was added with the pale red pyrene-NHS (10 mg, 0.0259 mmol) in 60 μL DMSO and the mixture was stored at room temperature for 20 hr until 1% lithium perchlorate in acetone (1 mL) was added to the reaction to result in white precipitation. The mixture was spun at 13k rpm for 10 min and the supernatant was removed. The pellet was washed with 1% lithium perchlorate in acetone (1 mL) twice and anhydrous ethanol (1 mL) three times. Then the pellet was dried in the air for a while before 300 μL autoclaved ddH_2O was added to make a stock solution of around 50 μM.

Additional measurements of standard solution

We detected Pb^{2+} concentration of standard solution with four devices and data are shown in Figure S2. There is a clear positive correlation between Pb^{2+} concentration and ΔVcnp in all devices. But there are obvious differences among each other, indicating the poor uniformity of our devices.

.

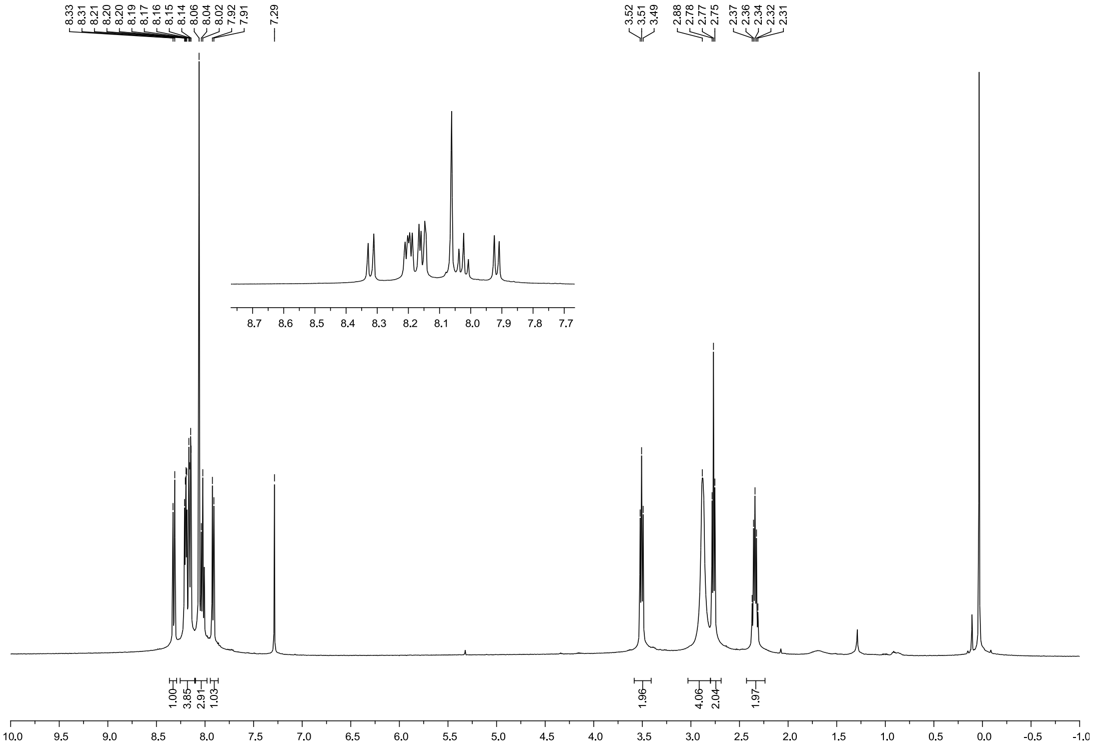

Figure S1 ^{1}H NMR spectrum of pyrene-NHS

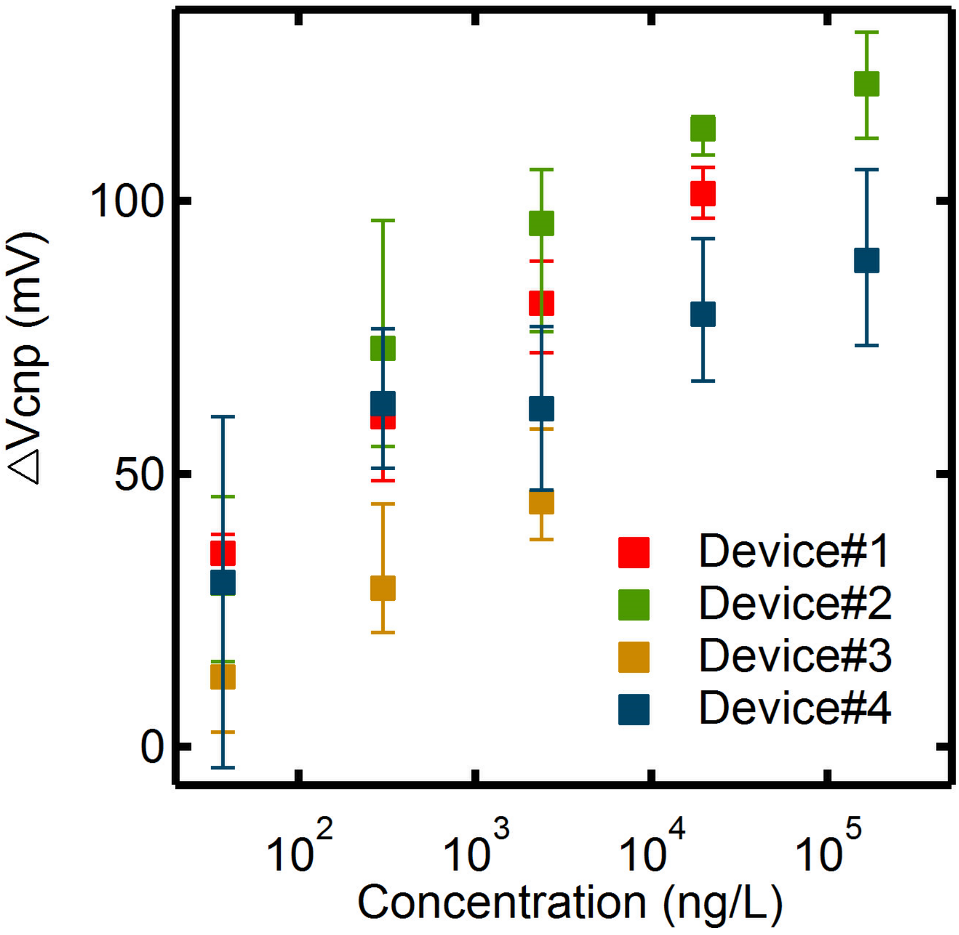

Figure S2 Standard solution detected by four devices
